# Supplementary material for: Disorder of consciousness rather than complete Locked-In Syndrome for end stage Amyotrophic Lateral Sclerosis: a case series
Source: Commun Med (Lond). 2025 Nov 19;5:482. doi: 10.1038/s43856-025-01196-9 (PMC12630916; doi:10.1038/s43856-025-01196-9)

Supplementary figures

Disorder of consciousness rather than complete Locked-In Syndrome for end stage Amyotrophic Lateral Sclerosis: A case series

F. Gobert[1,2], I. Merida[3*], E. Maby[4*], P. Seguin[4], J. Jung[4,5], D. Morlet[4], N. André-Obadia[5], F. Dailler[1], Ch. Berthomier[6], A. Otman[4]; D. Le Bars[3,7]; Ch. Scheiber[8]; A. Hammers[9,10], E. Bernard[11], N. Costes[3], R. Bouet[4] and J. Mattout[4]

<u>Supplementary Figure 1:</u> ***EEG reactivity during wake period***

A – Patient 1 at T_1: Alpha background rhythm with acceleration to the sensory stimulus “touching right arm” (blue line, “on lui touche le bras dt”).

B – Patient 2: Mixed frequency background rhythm with acceleration to the auditory stimulus “calling the patient by his name” (blue line, “appel nom prénom”).

EEG: bipolar montage, pages of 20 seconds, filters = 0.053 – 60 Hz, amplitude = 50 microV/cm.

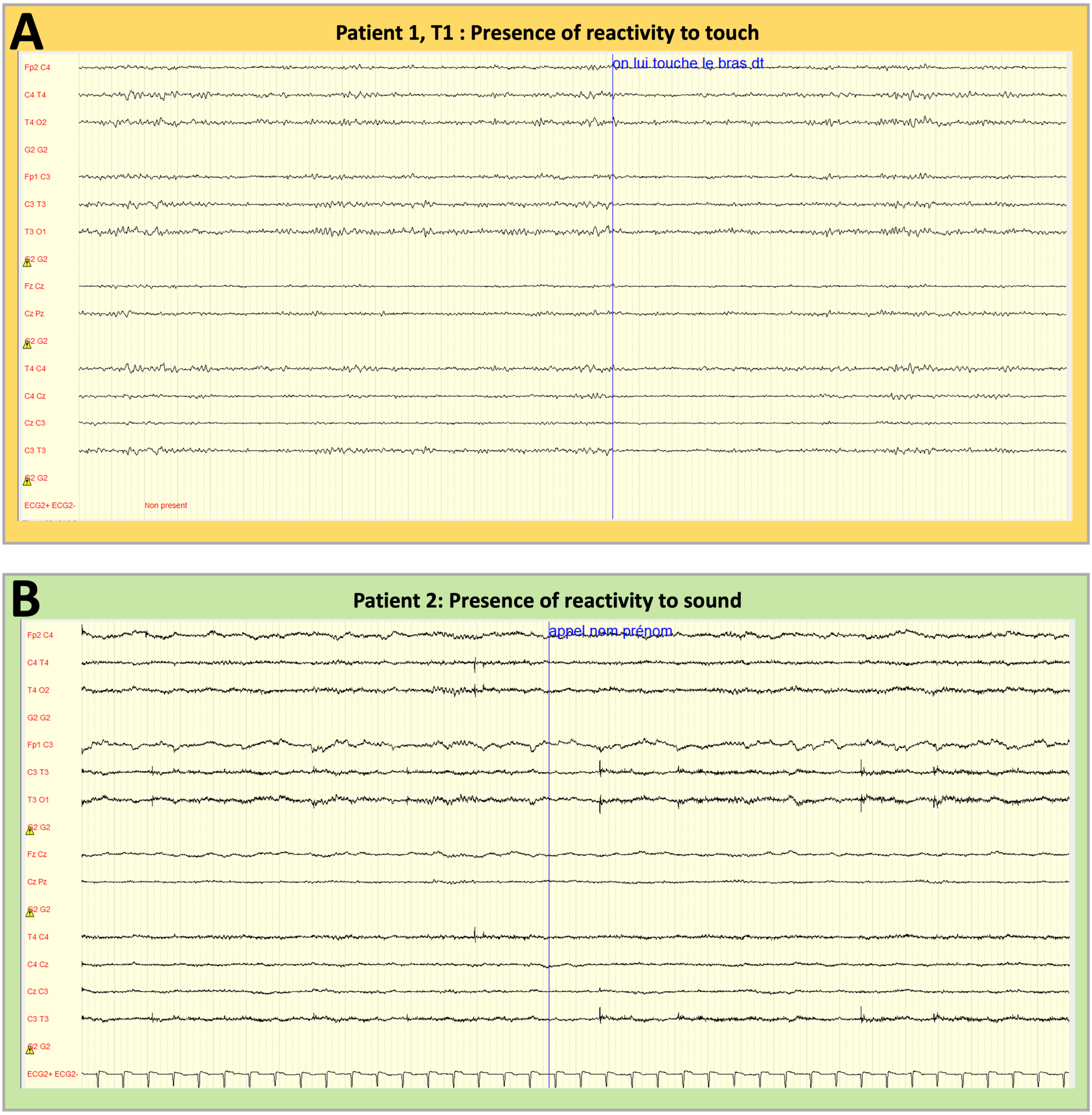

Supplementary Figure 2: ***Complement of PET analysis***

A – Patient 1: For PET illustration, the results are projected on the mean PET image of healthy subjects (in absence of MRI)

B – Patient 2: For PET illustration, the results are projected the co-acquired MRI.

Top part: [^{18}F]FDG-PET contrast “Patient < Healthy subjects” using an analysis of variance with 2 co-variables (on SUV images, uncorrected p<0.01) describing relative hypometabolism.

Bottom part: [^{18}F]F DG-PET contrast “Patient > Healthy subjects” using an analysis of variance with 2 co-variables (on SUV images, uncorrected p<0.01) describing relative hypermetabolism.

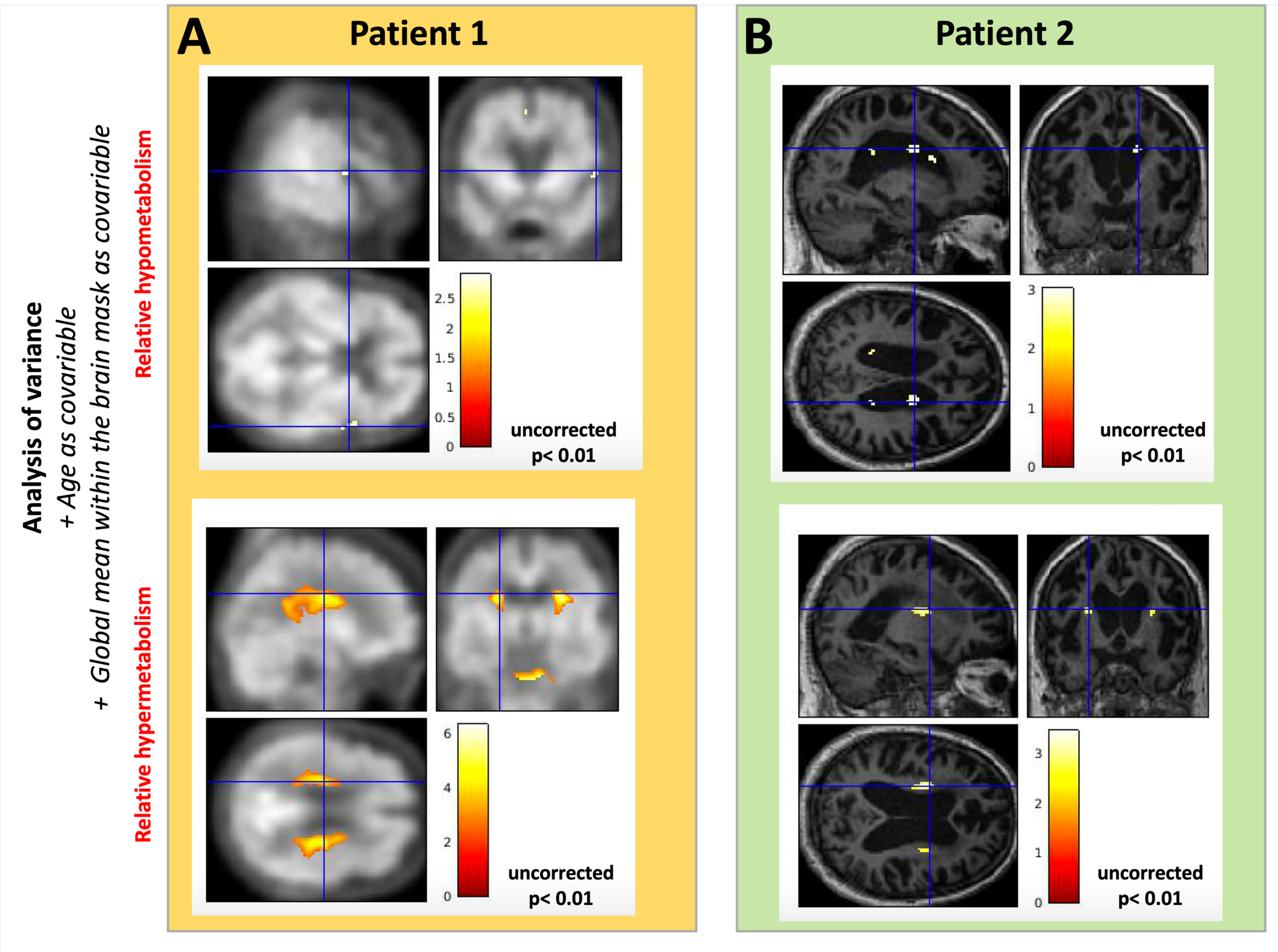

Supplementary Figure 3: ***Mental imagery task***

A – Patient 1: $[^{15}O]H_2O$ PET on PET-CT contrasting "Active (spatial navigation) – Passive (Rest) conditions using a SISCOM analysis (no significant difference)

B – Patient 2: Functional MRI on PET-MR contrasting "Active (spatial navigation) – Passive (Rest) conditions using a BOLD signal contrast (no significant difference at p-value of < 0.05 with FWE-correction).

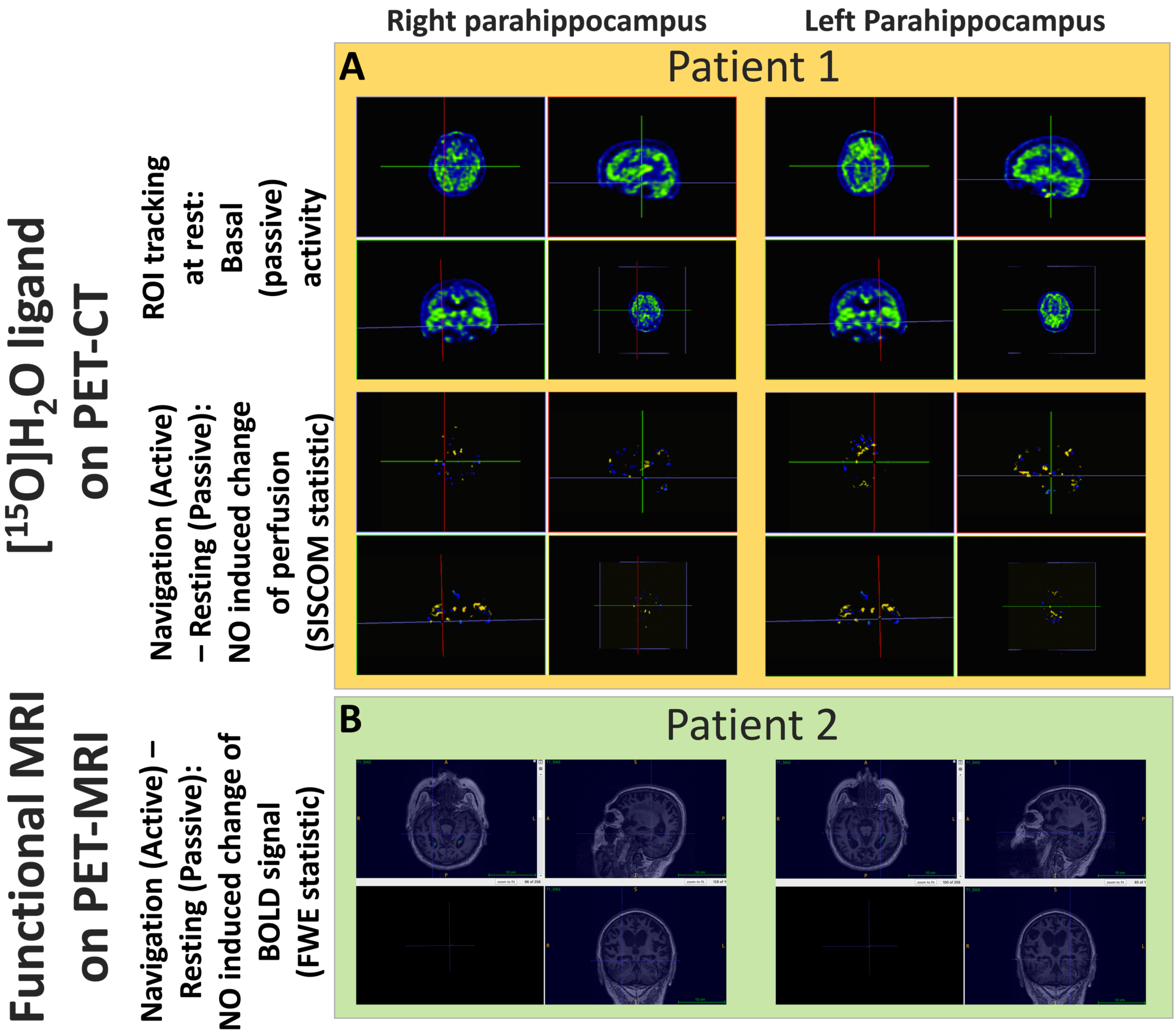

Supplement: Supplementary file 2 — Supplementary Information [file 43856_2025_1196_MOESM2_ESM.pdf]
