# Supplementary material for: Disorder of consciousness rather than complete Locked-In Syndrome for end stage Amyotrophic Lateral Sclerosis: a case series
Source: Commun Med (Lond). 2025 Nov 19;5:482. doi: 10.1038/s43856-025-01196-9 (PMC12630916; doi:10.1038/s43856-025-01196-9)
Supplement: Supplementary file 3 — Reporting Summary [file 43856_2025_1196_MOESM3_ESM.pdf]

nature portfolio

Corresponding author(s): Florent Gobert

Last updated by author(s): Aug 16, 2025

Reporting Summary

Nature Portfolio wishes to improve the reproducibility of the work that we publish. This form provides structure for consistency and transparency in reporting. For further information on Nature Portfolio policies, see our Editorial Policies and the Editorial Policy Checklist.

Statistics

For all statistical analyses, confirm that the following items are present in the figure legend, table legend, main text, or Methods section.

n/a	Confirmed	
☐	☒	The exact sample size (*n*) for each experimental group/condition, given as a discrete number and unit of measurement
☒	☐	A statement on whether measurements were taken from distinct samples or whether the same sample was measured repeatedly
☒	☐	The statistical test(s) used AND whether they are one- or two-sided<br>*Only common tests should be described solely by name; describe more complex techniques in the Methods section.*
☐	☒	A description of all covariates tested
☐	☒	A description of any assumptions or corrections, such as tests of normality and adjustment for multiple comparisons
☐	☒	A full description of the statistical parameters including central tendency (e.g. means) or other basic estimates (e.g. regression coefficient) AND variation (e.g. standard deviation) or associated estimates of uncertainty (e.g. confidence intervals)
☐	☒	For null hypothesis testing, the test statistic (e.g. F, t, r) with confidence intervals, effect sizes, degrees of freedom and P value noted<br>*Give P values as exact values whenever suitable.*
☒	☐	For Bayesian analysis, information on the choice of priors and Markov chain Monte Carlo settings
☐	☐	For hierarchical and complex designs, identification of the appropriate level for tests and full reporting of outcomes
☒	☐	Estimates of effect sizes (e.g. Cohen's *d*, Pearson's *r*), indicating how they were calculated

Our web collection on statistics for biologists contains articles on many of the points above.

Software and code

Policy information about availability of computer code

Data collection: No data collection available.

Data analysis: The code used for imaging data analysis (excluding BCI-related code) is also available from the corresponding author upon reasonable request.

For manuscripts utilizing custom algorithms or software that are central to the research but not yet described in published literature, software must be made available to editors and reviewers. We strongly encourage code deposition in a community repository (e.g. GitHub). See the Nature Portfolio guidelines for submitting code & software for further information.

Data

Policy information about availability of data

All manuscripts must include a data availability statement. This statement should provide the following information, where applicable:

- Accession codes, unique identifiers, or web links for publicly available datasets
- A description of any restrictions on data availability
- For clinical datasets or third party data, please ensure that the statement adheres to our policy

Anonymized imaging and neurophysiological data (excluding clinical data) are available from the corresponding author upon reasonable request. No public data repository has been used. As the figures presented are purely illustrative and do not contain numerical graphs, no source data are available.

Human research participants

Policy information about studies involving human research participants and Sex and Gender in Research.

Reporting on sex and gender	No selection based on such criteria.
Population characteristics	Pathological context of end-stage ALS
Recruitment	Based on the admission in our centre with such mechanism
Ethics oversight	Authorization was obtained from the ethics committee of the Hospices Civils de Lyon (Comité Scientifique et Éthique des Hospices Civils de LYON CSE-HCL – IRB 00013204; Pr Cyrille Confavreux; approval N. 24-310)

Note that full information on the approval of the study protocol must also be provided in the manuscript.

Field-specific reporting

Please select the one below that is the best fit for your research. If you are not sure, read the appropriate sections before making your selection.

☒ Life sciences ☐ Behavioural & social sciences ☐ Ecological, evolutionary & environmental sciences

For a reference copy of the document with all sections, see nature.com/documents/nr-reporting-summary-flat.pdf

Life sciences study design

All studies must disclose on these points even when the disclosure is negative.

Sample size	2
Data exclusions	No
Replication	No
Randomization	No
Blinding	No

Reporting for specific materials, systems and methods

We require information from authors about some types of materials, experimental systems and methods used in many studies. Here, indicate whether each material, system or method listed is relevant to your study. If you are not sure if a list item applies to your research, read the appropriate section before selecting a response.

Materials & experimental systems

n/a	Involved in the study	
☒	☐	Antibodies
☒	☐	Eukaryotic cell lines
☒	☐	Palaeontology and archaeology
☒	☐	Animals and other organisms
☐	☒	Clinical data
☒	☐	Dual use research of concern

Methods

n/a	Involved in the study	
☒	☐	ChIP-seq
☒	☐	Flow cytometry
☐	☒	MRI-based neuroimaging

Clinical data

Policy information about clinical studies

All manuscripts should comply with the ICMJE guidelines for publication of clinical research and a completed CONSORT checklist must be included with all submissions.

Clinical trial registration	No
Study protocol	No

Data collection	No
Outcomes	One patient is described twice.

Magnetic resonance imaging

Experimental design

Design type	Observation
Design specifications	Case series
Behavioral performance measures	CRS-R

Acquisition

Imaging type(s)	T1 T2
Field strength	3 T
Sequence & imaging parameters	functionnal, T2, FLAIR, T1
Area of acquisition	Whole Brain

Diffusion MRI ☐ Used ☒ Not used

Preprocessing

Preprocessing software	SPM
Normalization	SPM
Normalization template	No
Noise and artifact removal	No
Volume censoring	No

Statistical modeling & inference

Model type and settings	Statistical analyses of the data were conducted at the individual level to answer clinical questions. The results are qualitatively discussed at the phenotypic description level.<br>PET: SPM 12 general linear model<br>BCI: Bayesian classifier computed the posterior probability for each class given the observed features ("yes" or "no" target stimuli versus "yes" or "no" non-target stimuli). Then the output of each classifier was optimally combined to obtain a final and unique posterior probability for each of the two classes ("yes" or "no"). Complementary offline analyses were performed to assess BCI performance based on different stimulus types (standard, deviant, yes, no).
Effect(s) tested	*Define precise effect in terms of the task or stimulus conditions instead of psychological concepts and indicate whether ANOVA or factorial designs were used.*

Specify type of analysis: ☒ Whole brain ☐ ROI-based ☐ Both

Statistic type for inference (See Eklund et al. 2016)	*Specify voxel-wise or cluster-wise and report all relevant parameters for cluster-wise methods.*
Correction	FWE for PET

Models & analysis

n/a	Involved in the study
☒	☐ Functional and/or effective connectivity
☒	☐ Graph analysis
☐	☒ Multivariate modeling or predictive analysis

Multivariate modeling and predictive analysis	Multimodal battery: neurophysiological testing, passive and active auditory oddball paradigms, auditory Brain-Computer Interface (BCI), functional activation-task imaging, long-term EEG, brain morphology, and resting-state metabolism
